# Repeated bedside echocardiography in children with respiratory failure

**DOI:** 10.1186/1476-7120-9-14

**Published:** 2011-04-26

**Authors:** Jiri Kobr, Jiri Fremuth, Katerina Pizingerova, Lumir Sasek, Petr Jehlicka, Sarka Fikrlova, Zdenek Slavik

**Affiliations:** 1 Department of Paediatrics, Charles University in Prague, Faculty of Medicine in Pilsen and Faculty Hospital Pilsen, Czech Republic; 2 Department of Paediatrics, Paediatric Intensive Care Unit, Faculty Hospital Pilsen, Czech Republic; 3 Department of Cardiology, Paediatric Intensive Care Unit, The Royal Brompton Hospital NHS Trust, London, UK

## Abstract

**Background:**

The aim of this study was to verify the benefits and limitations of repeated bedside echocardiographic examinations in children during mechanical ventilation. For the purposes of this study, we selected the data of over a time period from 2006 to 2010.

**Methods:**

A total of 235 children, average age 3.21 (SD 1.32) years were included into the study and divided into etiopathogenic groups. High-risk groups comprised: Acute lung injury and acute respiratory distress syndrome (ALI/ARDS), return of spontaneous circulation after cardiopulmonary resuscitation (ROSC), bronchopulmonary dysplasia (BPD), cardiomyopathy (CMP) and cardiopulmonary disease (CPD). Transthoracic echocardiography was carried out during mechanical ventilation. The following data were collated for statistical evaluation: right and left ventricle myocardial performance indices (RV MPI; LV MPI), left ventricle shortening fraction (SF), cardiac output (CO), and the mitral valve ratio of peak velocity of early wave (E) to the peak velocity of active wave (A) as E/A ratio. The data was processed after a period of recovery, i.e. one hour after the introduction of invasive lines (time-1) and after 72 hours of comprehensive treatment (time-2). The overall development of parameters over time was compared within groups and between groups using the distribution-free Wilcoxons and two-way ANOVA tests.

**Results:**

A total of 870 echocardiographic examinations were performed. At time-1 higher average values of RV MPI (0.34, SD 0.01 vs. 0.21, SD 0.01; p < 0.001) were found in all groups compared with reference values. Left ventricular load in the high-risk groups was expressed by a higher LV MPI (0.39, SD 0.13 vs. 0.29, SD 0.02; p < 0.01) and lower E/A ratio (0.95, SD 0.36 vs. 1.36, SD 0.64; p < 0.001), SF (0.37, SD 0.11 vs. 0.47, SD 0.02; p < 0.01) and CO (1.95, SD 0.37 vs. 2.94, SD 1.03; p < 0.01). At time-2 RV MPI were lower (0.25, SD 0.02 vs. 0.34, SD 0.01; p < 0.001), but remained higher compared with reference values (0.25, SD 0.02 vs. 0.21, SD 0.01; p < 0.05). Other parameters in high-risk groups were improved, but remained insignificantly different compared with reference values.

**Conclusion:**

Echocardiography complements standard monitoring of valuable information regarding cardiac load in real time. Chest excursion during mechanical ventilation does not reduce the quality of the acquired data.

## Background

The severity of a patient's clinical condition is proportional to the quality of blood circulation, changes in cardiac loading conditions, and global myocardial performance. Relevant and easily interpretable information regarding the current hemodynamics is essential for effective and safe therapeutic manipulation of blood circulation. The issue of non-invasive hemodynamic monitoring of critically ill patients using echocardiography has been extensively studied [1-4]. The WINFOCUS experience has determined the ideal conditions for intensivists training and obtaining accreditation for the use of echocardiography in intensive care [5]. The conclusions of this study warrant the extension and application of echocardiographic techniques in this setting.

At our centre we prefer minimally invasive techniques to collect clinically useful hemodynamic data. Since 1997, we utilize bedside echocardiography as an integral component of established comprehensive care of critically ill children.

Echocardiographic views and measurements were carried out as recommended by ASE/EAE [6]. For the purposes of this study, we selected the data of consecutive patients with respiratory failure over a time period from 2006 to 2010. All children were investigated during mechanical ventilation, i.e. non-standard conditions.

## Aim of the study

We aimed to verify the benefits and limitations of repeated bedside echocardiographic examinations in children during mechanical ventilation.

## Methods

All patients included in the study were subjected to full history taking, physical examination, routine investigations, twelve lead ECG and chest X-ray. All children were monitored for vital functions, i.e. heart and respiratory rate, pulse oxymetry, ECG and one-hour diuresis. Systemic arterial and venous pressures were invasively measured with vascular catheters. The first bedside echocardiographic evaluations of cardiac performance were performed on patient admission to the PICU, and then repeated according to clinical urgency. Symptoms of severe circulatory compromise and reduction in systemic pressures were indications for further detailed investigations. In such cases, patients were examined using detailed echocardiographic techniques in a specialized laboratory using a GE Vivid 7 ultrasound apparatus (GPS Medical; USA). Hemodynamics were monitored simultaneously using other minimally invasive techniques such as Vigileo (Edwards; USA) or Uscom (Spacelabs Healthcare; Australia).

Protective conventional mechanical lung ventilation was conducted based on the principle of positive pressure ventilation [7]. Non-conventional forms of ventilation were utilized: high-frequency oscillatory ventilation, exogenous surfactant replacement, inhalation of nitric oxide for selective vasodilatation or tracheal gas insufflation to eliminate carbon dioxide. Mechanical lung ventilation was accompanied by volume treatment, parenteral nutrition and pharmacological support of blood circulation and diuresis.

### Distribution of patients and data collection

According to the prevailing etiopathogenesis of respiratory failure, patients were divided into eight groups and all data was evaluated in these groups. Etiopathogenetic groups comprised of acute lung injury patients and those with acute respiratory distress syndrome (ALI/ARDS), asthmatic condition, bronchiolitis acuta (BOA), return of spontaneous circulation after cardiopulmonary resuscitation (ROSC), bronchopulmonary dysplasia (BPD), cardiomyopathy (CMP), cardiopulmonary disease (CPD), severe sepsis and septic shock according to the Society of Critical Care Medicine (Sepsis).

Data was assessed at two separate intervals: after a period of recovery, i.e. one hour after the introduction of invasive lines (time-1) and after 72 hours of comprehensive treatment (time-2).

For evaluation of cardiac load and myocardial performance during mechanical ventilation, we chose the following echocardiographic parameters: Right ventricle myocardial performance index (RV MPI), left ventricle myocardial index (LV MPI), left ventricle shortening fraction (SF), cardiac output (CO; l/min.) and mitral valve ratio of the peak velocity of early wave (E; cm/s) to the peak velocity of active wave (A; cm/s) to quantitative evaluation of the left ventricle mechanics (E/A ratio).

### Echocardiography

Patients during mechanical ventilation were investigated at their bedside using transthoracic echocardiography (TTE). The TTE examination was performed using commercially available MicroMaxx (SonoSite Inc.; USA) with high-resolution 3.5-5.5 MHz sequential transducer equipment and calculation package. M-mode, two dimmensional (2D), spectral flow and color Doppler imaging were performed according to the consensus recommendations of professional societies [8].

**Figure 1:**
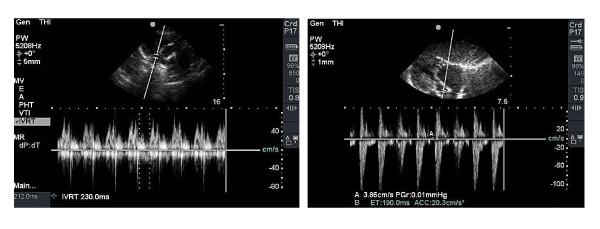
**Mitral valve inflow, left ventricle filling and outflow**.

M-mode and 2D examinations were performed in standard axis modes [9]. The left ventricular internal dimensions and wall thickness were measured in M-mode. The SF, EF and CO were calculated using the ultrasound device. 2D echocardiographic views of standard projections were used for anatomical orientation and to find structural defects. Continuous spectral Doppler flow with dual color Doppler imaging was used to explore for regurgitation, valves or septal defects, and the measurement of peak pressure gradients (PPGs; torr). Data from PPG tricuspid valve regurgitation and systemic pressures were used to manually calculate the mean pressure in the right ventricle. This corresponds to a mean pressure in the pulmonary trunk, unless an obstruction of right ventricular outflow tract or pulmonary regurgitation exists. Pulsed Doppler flow with high resolution was used to measure the velocity of atrioventricular valve inflow and the time interval between valvular closure and opening. The apical four-chamber view enables us to acquire data of blood inflow through the atrioventricular valves, and in a left parasternal view of the semilunar valves inflow. The data obtained were used to calculate E/A ratio and MPIs (Tei-indices). Care was taken to align the transducer beam as closely as possible to the blood flow axis. Doppler signals for the left and right ventricular valves were not acquired simultaneously. No angle correction was made. Doppler and ECG tracings were recorded and stored digitally. The blood flow time intervals were measured by taking the three most distinct Doppler traces in a frozen template. The time interval from the cessation to the onset of mitral or tricuspid inflow (AVCO; ms) was measured. This interval is equal to the sum of isovolumic relaxation time (IRT; ms), isovolumic contraction time (ICT; ms), and ejection time (ET; ms). The actual view of the mitral valve inflow and measurement of time intervals of the left ventricle filling and outflow are shown in Figure 1.

Left and right ventricular ejection time was measured in the ascending aorta or pulmonary trunk just above the corresponding valve. Myocardial performance index (MPI) was then calculated using MPI = (AVCO - ET) / ET selectively for the right (RV MPI) and left (LV MPI) heart ventricles [10,11]. The elevation values of the MPIs corresponding reduction in global, i.e. systolic and diastolic ventricular function. Thus the calculated value was divided by the current value of the R-R interval of simultaneously recorded electrocardiogram to exclude the impact of variations in heart rate on the measurement result. This method was used to calculate the myocardial performance index for each of the three measurements.

The average value of these three indices was stored in a database. This data was used for statistical processing.

### Statistical analysis

All measured parameters were presented as a mean with standard deviation (mean ± SD). For qualitative analysis and a comparison of the distribution of studied parameters of groups, a Student's t-test and distribution-free Wilcoxons test applied. The overall development of studied parameters over time was compared between both groups using the two-way ANOVA test. A level of statistical significance of *P*< 0.05 was accepted. All the data were analyzed using statistical software (Analyze-it 211 Software Ltd.).

## Results and Discussion

The study included a total of 235 children. Their clinical characteristics on admission are presented in Table 1.

**Table 1:** Clinical characteristics of the study population (n = 235)

*Characteristics*	*Values*	*Units*
Age	3.21 ± 1.32	Years; mean ± SD
Gender male/female	47 / 53	%
Body weight	15.72 ± 2.75	Kg; mean ± SD
PRISM score	15.36 ± 9.63	Points; mean ± SD
Predominant etiology of respiratory failure		
Pulmonary	168; (71.5)	N; (%)
Cardiogenic	32; (13.6)	N; (%)
Septic	26; (11.1)	N; (%)
Other	9; (3.8)	N; (%)

A total of 870 echocardiographic examinations were performed during the study. In addition, we also performed an additional 60 echocardiography examinations in 60 hemodynamically stable children, in order to obtain reference values of the parameters: RV MPI (0.21 ± 0.01), LV MPI (0.29 ± 0.02), SF (0.47 ± 0.02), CO (2.94 ± 1.03) and E/A ratio (1.36 ± 0.64). Reference data values of the MPIs were in agreement with the reference values of British authors [2].

We assume that the update MPIs of our calculations by parallel R-R interval of ECG was the cause of our lower reference data (RV MPI 0.21 ± 0.011 vs. 0.27 ± 0.09; p < 0.01 and LV MPI 0.29 ± 0.015 vs. 0.32 ± 0.07; p < 0.05) compared with MPI data of other studies [12].

Distribution of patients in the etiopathogenetic groups, their basic characteristics, presence of structural heart defects, functional cardiac abnormalities and the ratio of actual to predicted mortality in the groups are listed in Table 2.

**Table 2:** Distribution of patients into groups, echocardiographic findings and actual mortality

*Etiology*	*N*	*Age*	*Exam/patient*	*Structural defects*	*Functional abnormalities*	*RAPM ratio*
ALI/ARDS	103	1.08 ± 0.34	14.8 ± 1.23	7	35	0.58 ± 0.02
Asthma	8	5.70 ± 2.66	8.7 ± 1.77	0	1	0
BOA	53	1.82 ± 1.06	11.1 ± 1.48	2	7	0.53 ± 0.01
BPD	11	0.55 ± 0.22	17.6 ± 3.12	5	8	0.67 ± 0.03
ROSC	12	3.70 ± 1.12	17.7 ± 3.03	4	9	0.76 ± 0.01
CMP	9	4.79 ± 2.75	10.6 ± 2.36	0	6	0.61 ± 0.06
CPD	13	5.56 ± 1.38	12.9 ± 1.64	11	13	0.51 ± 0.04
Sepsis	26	2.47 ± 1.01	16.2 ± 0.97	2	7	0.46 ± 0.02

*Summary*	235	3.21 ± 1.32	13.7 ± 1.95	33	74	0.56 ± 0.02

N: number of patients in group, Age: average age in years ± SD; Exam/patient, the number of echocardiographic examinations per patient throughout the hospitalization, measured as mean ± SD, Structural defects: total number of structural congenital heart defects (i.e. ventricular outflow or inflow obstructions, left-right or right-left shunts, critical, complex and combined defects), Functional abnormalities: total number of functional abnormalities (tricuspid valve regurgitation > 15 mmHg or/and mitral regurgitation > 10 mmHg), RAPM ratio: Ratio of Actual to Predicted Mortality expressed as mean ± SD.

The most affected patients were in groups ALI/ARDS, BPD, ROSC, CMP, and CPD. These populations can be described as high-risk groups with highest real mortality (RAMP ratio). Not surprisingly, the CPD group was identified as having the highest number of structural congenital heart defects with significant left-right shunt, such as ventricular septal defects, atrioventricular septal defects, patent arterial duct or common arterial trunk. The average value of the PPG tricuspid valve regurgitation was 23.63 ± 2.55 torr and mitral regurgitation 10.42 ± 1.67 torr.

An overview of data obtained at time-1 is presented in Table 3.

**Table 3:** Mean values with standard deviations for echocardiographic data at time-1

*Groups*	RV MPI	LV MPI	SF	CO	E/A ratio
ALI/ARDS	0.38 ± 0.19	0.37 ± 0.18	0.41 ± 0.12	1.97 ± 0.66	1.10 ± 0.44
Asthma	0.36 ± 0.15	0.35 ± 0.17	0.37 ± 0.09	2.32 ± 0.83	1.26 ± 0.63
BOA	0.33 ± 0.12	0.36 ± 0.14	0.40 ± 0.11	1.99 ± 0.28	1.33 ± 0.45
BPD	0.33 ± 0.09	0.37 ± 0.03	0.39 ± 0.10	2.21 ± 0.20	0.92 ± 0.17
ROSC	0.29 ± 0.06	0.41 ± 0.12	0.31 ± 0.12	1.58 ± 0.32	0.78 ± 0.34
CMP	0.32 ± 0.14	0.41 ± 0.15	0.35 ± 0.11	1.92 ± 0.26	0.96 ± 0.35
CPD	0.36 ± 0.15	0.37 ± 0.17	0.39 ± 0.08	2.07 ± 0.41	1.00 ± 0.50
Sepsis	0.32 ± 0.22	0.37 ± 0.17	0.34 ± 0.13	1.99 ± 0.24	1.04 ± 0.21

**Figure 2:**
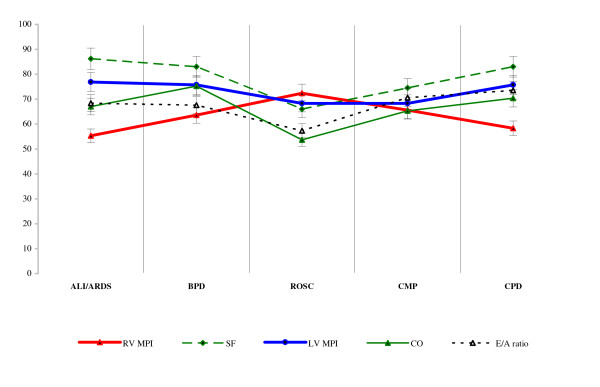
**Percentages of data within high-risk groups at time-1**.

Data obtained at time-1 in the high-risk groups was expressed for greater clarity as a percentage of reference values in Figure 2.

At the beginning of the study, i.e. at time-1 of the study, the RV MPI (0.21 ± 0.011 vs. 0.34 ± 0.13; p < 0.001) were higher in all etiological groups of critically ill children compared with reference values. In the high-risk groups there were also higher LV MPI (0.29 ± 0.015 vs. 0.39 ± 0.13; p < 0.001) and lower SF (0.47 ± 0.022 vs. 0.37 ± 0.11; p < 0.05), CO (2.94 ± 1.03 vs. 1.95 ± 0.37; p < 0.001) and E/A ratio (1.36 ± 0.64 vs. 0.95 ± 0.36; p < 0.01), compared with reference values. Within the high-risk groups SF (0.31 ± 0.12 vs. 0.50 ± 0.13; p < 0.001), CO (1.58 ± 0.32 vs. 2.27 ± 0.93; p < 0.001) and E/A ratio (0.78 ± 0.34 vs. 1.36 ± 0.27; p < 0.001) were lowest in the ROSC group. The findings correspond to the etiopathogenesis and clinical severity of this group.

Differences in data over time in each group were interesting. The differences within groups at time-2 and time-1 are presented in Table 4.

**Table 4:** Data differences observed within groups at time-2 and time-1

*Groups*	RV MPI *Values p <*	LV MPI *Values p <*	SF *Values p <*	CO *Values p <*	E/A ratio *Values p <*
ALI/ARDS	0.001	NS	0.05	0.05	0.01
Asthma	0.001	0.01	0.01	0.01	0.05
BOA	0.001	0.01	0.05	0.01	NS
BPD	0.01	0.05	NS	0.05	0.01
ROSC	NS	0.01	0.01	0.01	0.01
CMP	0.01	0.05	0.05	0.05	NS
CPD	0.001	0.05	0.05	0.05	0.05
Sepsis	0.05	0.05	0.01	0.05	0.05

**Figure 3:**
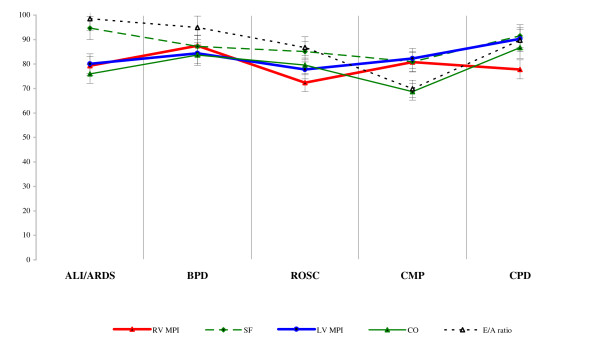
**Percentages of data within high-risk groups at time-2**.

The data obtained at time-2 in the high-risk groups was also graphically expressed as a percentage of reference values and presented in Figure 3.

The values of all monitored parameters had a positive trend in chronological order. After three days of intensive care, i.e. at time-2 of study, average RV MPI remained higher (0.23 ± 0.01 vs. 0.21 ± 0.01; p < 0.05) in all etiological groups compared with reference values. In the high-risk groups also remained higher average LV MPI (0.31 ± 0.02 vs. 0.29 ± 0.02; p < 0.05) and lower average CO (2.35 ± 0.47 vs. 2.94 ± 1.03; p > 0.05), compared with reference values. At time-2 there were the smallest differences in the CMP group. In this group the E/A ratio (0.95 ± 0.10 vs. 1.36 ± 0.33; p < 0.01), SF (0.38 ± 0.03 vs. 0.53 ± 0.11; p < 0.01) and the values of CO (2.02 ± 0.24 vs. 2.35 ± 0.47; p < 0.05) remained the lowest. The results suggested a low compliance and low left ventricular contractility in the CMP group compared with the results of other groups at time-2. In the ROSC group at time-2, values of LV MPI, SF, CO, and E/A ratio were found to be improved, but RV MPI (0.29 ± 0.02 vs. 0.25 ± 0.01; p < 0.05) remained the highest compared with the high-risk groups. The result corresponded to increased right ventricular afterload in this group.

Quantitative evaluation of data changes over time was informative. The difference between complete dataset obtained at time-2 and time-1 using the Wilcoxon paired test are presented in Table 5.

**Table 5:** Comparison of data at time-2 and time-1

*Parameters*	*Difference variables*	*Statistic*	*Alpha*	*Values p <*
RV MPI	-0.064	1009.5	0.00	0.001
LV MPI	-0.027	3714.0	0.42	0.001
SF	0.043	2188.5	0.00	0.001
CO	1.211	4152.3	0.29	0.01
E/A ratio	0.332	3022.7	0.56	0.05

The benefits of MPIs have been widely discussed in the literature. MPI values provide comprehensive information regarding systolic and diastolic ventricular function in real time [13-15].

The study demonstrated that a combination of respiratory failure and mechanical ventilation significantly increased the right ventricular loading conditions, regardless of the etiology of the disease. Since the beginning of our study, we evaluated the global performance of the right ventricle using RV MPI due to it being technically simpler. The variability of the RV MPI data set was 0.00316, sensitivity of RV MPI was 0.891, and specificity 0.814. To assess right ventricular systolic function it could be more useful to measure the tricuspid plane systolic annular excursion (TAPSE) [16]. This newer methodology however should be performed by a trained cardiologist in order to ensure standardization. Our methodology on the other hand can be performed by a trained intensivist.

On the contrary, LV MPI and CO varied according to the etiopathogenesis of disease in the other groups. The variability of the LV MPI data set was 0.00501, sensitivity of LV MPIs was 0.878, specificity 0.834. Normalization values of the monitored parameters and rate of change was dependent on the etiopathogenesis of the disease. Our results support the conclusions of other studies that determined a high degree of confidence between MPIs and cardiac loading conditions. Nevertheless the MPIs being dependent on loading conditions closely follow changes in myocardial function and appear to have predictive value mainly in myocardial dysfunction [17,18]. This probably reflects changes in isovolumic contraction time and ejection time most likely to be affected by disease states leading to myocardial systolic dysfunction and increased afterload. Treatment interventions altering myocardial systolic function and afterload with concomitant shortening of isovolumic contraction time and lengthening of ejection time will lead to improved values of the MPIs [19,20]. In line with the conclusions of several studies, we confirm that the monitoring of changes MPIs provides valuable information in real time [21-23]. The clinical assessment of each subject does not depend upon absolute values, but rather the trend of the measured parameters.

## Limitations

The authors are aware that echocardiography during mechanical ventilation has limitations related to methodology, parameter selection and investigator experience. Relative methodological limits of echocardiographic views are ventral pneumothorax, or left-sided alveolar hyperinflation. The presence of a larger volume of air between the ultrasound probe and the heart, lodged deep in the chest reduces image quality. A cardiologist using M-mode may reveal a ventral pneumothorax and is thus forced to seek non-standard projections for further examination of the heart. Modern ultrasound procedures enable high quality assessment of lung parenchyma under the B-line [24]. In such cases it is advantageous to use the subcostal view, which allows examination of acceptable quality. The subcostal view was helpful in evaluating patients during high frequency oscillatory ventilation. On the other hand, other very sophisticated parameters monitored during mechanical ventilation do not have the same reliability [25,26]. Choosing the optimal axis of the Doppler probe may be a limitation of the investigator, depending on his experience. Contrary measurement of the time interval between the opening and closing of the atrioventricular valves, however, is not investigator dependent. This measurement is imprecise during tachycardia where this interval is extremely shortened. We feel that any potential inaccuracies of this method are counterbalanced when the same investigator performs all the measurements in each subject. All the above limitations do not detract from the importance of the MPIs, left ventricular SF, CO and mitral valve E/A ratio as a noninvasive, easily obtainable, and reproducible tool for the assessment of cardiac performance in the setting of clinical intensive care. It should be emphasized that quality testing is for all methods of monitoring hemodynamics dependant on the hands-on investigator experience. Based on our experience and the experience of others serial measurements of these indices are likely to be a valuable tool in close monitoring of cardiac performance in critically ill patients.

## Clinical Implications

Repeated bedside echocardiography is a useful addition to the comprehensive hemodynamic monitoring of critically ill children. The absolute values of the measured parameters are not as important as the dynamic changes during the course of treatment.

## Conclusion

Breathing chest excursion during mechanical ventilation does not diminish the quality of echocardiography. Echocardiography complements monitoring of valuable information regarding the morphology and real-time changes in ventricular loading conditions. Repeated bedside examination enables the control of functional changes, current compliance and overall biventricular performance. Tracking changes in cardiac performance contributes towards the effectivity of treatment. Cardiac output and hemodynamics are affected by the etiology of the disease, management strategies and treatment time.

## List of abbreviations

ALI/ARDS: acute lung injury and acute respiratory distress syndrome; Asthma: asthmatic condition; AVCO: time interval from cessation to the onset of mitral or tricuspid inflow; BOA: bronchiolitis acuta; BPD: bronchopulmonary dysplasia; CO: cardiac output (according to the Teichholtz formula); CMP: cardiomyopathy; CPD: cardiopulmonary disease; E/A ratio: mitral valve ratio of peak velocity of the wave E to wave A.; LV MPI: left ventricle myocardial performance index; PRISM: Predicted Risk of Mortality Score (pediatric version); PICU: Paediatric Intensive Care Unit; PPG: peak pressure grading; RAPM ratio: Ratio of Actual to Predicted Mortality; ROSC: return of spontaneous circulation after successful cardiopulmonary resuscitation; RV MPI: right ventricle myocardial performance index; Sepsis: severe sepsis and septic shock (according to the Society of Critical Care Medicine); SF: left ventricle shortening fraction; TTE: transthoracic echocardiography.

## Competing interests

The authors declare that they have no competing interests.

## Authors' contributions

JK and ZS introduced the study idea. JK, JF and PJ acquired the echocardiography images, KP and LS helped in the interpretation of the results, and SF helped with data collection. JK wrote the manuscript, ZS and KP added clinical discussion to the manuscript. ZS reviewed the manuscript. Finally, all authors read and approved the manuscript.
